# Revealing novel *cytb* and *nad*5 genes-based population diversity and benzimidazole resistance in *Echinococcus granulosus* of bovine origin

**DOI:** 10.3389/fvets.2023.1191271

**Published:** 2023-06-16

**Authors:** Mughees Aizaz Alvi, Ayed Alshammari, Rana Muhammad Athar Ali, Shahbaz Ul Haq, Rizwan Bashir, Li Li, Muhammad Saqib, Muhammad Sohail Sajid, Muzafar Ghafoor, Muhammad Imran, Muhammad Umar Ijaz, Bao-Quan Fu, Mohd Saeed, Irfan Ahmad, You-Yu Liu, Hong-Bin Yan, Wan-Zhong Jia

**Affiliations:** ^1^State Key Laboratory for Animal Disease Control and Prevention, College of Veterinary Medicine, Lanzhou University, National Para-Reference Laboratory for Animal Echinococcosis, Lanzhou Veterinary Research Institute, Chinese Academy of Agricultural Sciences, Lanzhou, China; ^2^Department of Clinical Medicine and Surgery, University of Agriculture, Faisalabad, Pakistan; ^3^Department of Biology, College of Science, University of Hafr Al Batin, Hafr Al Batin, Saudi Arabia; ^4^Key Laboratory of Veterinary Pharmaceutical Development, Ministry of Agriculture and Rural Affairs, Lanzhou Institute of Husbandry and Pharmaceutical Sciences, Chinese Academy of Agricultural Sciences, Lanzhou, China; ^5^Veterinary Disease Diagnostic Laboratory Sialkot, Livestock and Dairy Development Department, Government of Punjab, Lahore, Pakistan; ^6^Department of Parasitology, University of Agriculture, Faisalabad, Pakistan; ^7^Department of Pathology, University of Agriculture, Faisalabad, Pakistan; ^8^Department of Zoology, Wildlife and Fisheries, University of Agriculture, Faisalabad, Pakistan; ^9^Department of Biology, College of Science, University of Hail, Hail, Saudi Arabia; ^10^Department of Clinical Laboratory Sciences, College of Applied Medical Sciences, King Khalid University, Abha, Saudi Arabia; ^11^Jiangsu Co-Innovation Center for Prevention and Control of Important Animal Infectious Disease, Yangzhou, China

**Keywords:** *Echinococcus granulosus*, genetic diversity, phylogeny, benzimidazole resistance, *cytb*, *nad5*

## Abstract

Cystic echinococcosis (CE) is a neglected zoonotic disease caused by *Echinococcus granulosus* (*sensu stricto*). The parasite affects a wide range of livestock and wild animals. In this study, the population diversity of the *Echinococcus* species was investigated based on mitochondrial cytochrome b (*cyt*b) and NADH dehydrogenase subunit 5 (*nad*5) genes. In addition to this, β-tubulin gene isoforms of *Echinococcus granulosus* were amplified to determine the resistance against benzimidazoles. For this purpose, 40 cyst samples from cattle (*n* = 20) and buffaloes (*n* = 20) were collected from the main abattoir of Sialkot. DNA extraction was performed using Qiagen Blood and Tissue Kits. Amplification was performed through PCR. Each amplicon was confirmed by GelRed™ stained agarose gel (2%). Samples were sequenced in a DNA analyzer and viewed for any misread nucleotide by using MEGA (v.11). Corrections in nucleotide sequence and multiple sequence alignment were made through the same software. NCBI-BLAST was used for sample specific sequences to identify them as belonging to a particular species. Diversity indices were estimated using DnaSP (v.6) while phylogenetic analysis was inferred using the Bayesian method using MrBayes (v.1.1). β-tubulin gene isoforms sequence analysis was performed to find out the candidate gene causing benzimidazole resistance. All 40 isolates were found positive for *E. granulosus.* BLAST-based searches of sequences of each isolate for each gene (*nad*5 and *cyt*b) confirmed their maximum similarity with the G1 genotype. Overall, high haplotype diversity (Hd *nad5* = 1.00; Hd *cytb* = 0.833) and low nucleotide diversity (*π nad5* = 0.00560; *π = cytb* = 0.00763) was identified based on diversity indices. For both the genes, non-significant values of Tajima’s D (*nad*5 = −0.81734; *cyt*b = −0.80861) and Fu’s Fs (*nad*5 = −1.012; *cyt*b = 0.731) indicate recent population expansion. Bayesian phylogeny-based results of *nad5* and *cytb* sequences confirmed their genotypic status as distinct from other *Echinococcus* species. This study shed light on the status of benzimidazole resistance in *Echinococcus granulosus* for the very first time from Pakistan. The findings of this study will significantly add in the information available on genetic diversity of *Echinoccous granulosus* based on *cytb* and *nad5* genes sequences.

## 1. Introduction

Pakistan is an agriculture-dependent country heavily relying on the livestock sector which contributes 60.03% in agriculture and plays an important role in uplifting the national economy by accounting for 11.53% of the national gross domestic product (GDP) (1). A recent livestock census revealed that there are at least 51.5 million and 42.4 million heads of cattle and buffalo in the country, respectively (1). Several worldwide reports suggest that parasitic diseases cause significant economic losses to the livestock industry and severely affect weight gain, feed conversion efficacy, and the health of the animals. Among these parasitic diseases, potential harms contributed by *Echinococcus* infection are being neglected; therefore, cystic echinococcosis (CE) was listed in a subcategory of selected neglected tropical diseases (NTDs) to be addressed by the World Health Organization’s action plan to control NTDs (2, 3). The reported prevalence in Pakistan of the *Echinococcus* species in cattle, buffalo, sheep, goat, and camel is 5, 7, 7.5, 5, and 17%, respectively (4).

The *Echinococcus granulosus sensu* lato complex includes at least five cryptic species and some distinct genotypes namely *E. granulosus sensu stricto* (G1, G3), *E*. *equinus* (G4), *E*. *ortleppi* (G5), *E. canadensis* (G6-8, G10), and *E*. *felidis* (5–7). Cystic echinococcosis (CE) is transmitted between carnivores and herbivore/omnivore species which serve as definitive and intermediate hosts, respectively. *Echinococcus granulosus sensu stricto* (s.s.) possesses a wide intermediate host range (including bovine) which bear parasite larval stages in different visceral organs (8). This neglected zoonosis is a disease of public health concern and has serious economic setbacks. *E. granulosus* poses significant threats to human and animal health besides significant economic losses (9). Cosmopolitan distribution of this ailment had led to losses of USD 3 billion annually (10).

Resistance to benzimidazole drugs, an inherited genetic trait in parasites, has been observed worldwide and it appears to be enhancing (11). It is associated with nucleotide substitution in the β-tubulin gene, which indicates conservative point mutations. Anthelmintic resistance allele identification is critical for understanding the mechanisms involved and the epidemiology of anthelmintic resistance. The lack of data on these aspects of disease put livestock and human populations at high risk and may result in a high burden of disease in the future.

Owing to the limited data on the population structure of CE especially based on *nad*5 and *cyt*b genes, and with no research on benzimidazole resistance having been conducted in Pakistan, the present study was designed to determine genetic diversity and benzimidazole resistance in *Echinococcus granulosus* recovered from cattle and buffalo in Pakistan.

## 2. Materials and methods

### 2.1. Sample collection, processing, and DNA extraction

This study was carried out in the Sialkot district of the Punjab province of Pakistan. Twenty cyst samples were taken from cattle and another 20 from buffalo from the main abattoir under the jurisdiction of the municipal corporation of Sialkot from male as well as female animals during January–April, 2022. After slaughtering, carcasses were examined thoroughly for the presence of *Echinococcus* cysts particularly in the liver, lungs, and kidneys through visual inspection and palpation. Collected samples were delivered to the Department of Clinical Medicine and Surgery, Faculty of Veterinary Science, University of Agriculture, Faisalabad, Pakistan, under proper refrigerated conditions and processed aseptically. Ethical approval/consent from the animal owners was not required as no living animal was included in the study and the samples for this study were taken from the condemned carcasses of slaughtered animals.

After samples were delivered to the laboratory, each cyst was handled carefully. Each cyst was washed multiple times with a normal saline solution to decrease the chances of contamination. Then each cyst was washed with 70% ethanol. Cyst contents including fluid and protoscoleces were collected using sterile syringes and placed in conical 50 mL tubes (SPL Life Science Co., Ltd.). Then, an incision was made on the cyst wall and the remaining protoscoleces and cyst fluid were collected. The germinal membrane was taken from the cyst not apparently having protoscoleces at the bottom. Cyst contents were shifted to the sterile test tubes for centrifugation at 3,000 rpm at room temperature for 10 min. The supernatant was removed and only 1.5 mL of the precipitate from the bottom of the test tube was collected in non-pyrogenic, 1.5 mL microcentrifuge tubes (Kirgen, Solutions for Science). DNA was extracted from the germinal layer and/or collected protoscoleces using Qiagen Blood and Tissue Kits (Qiagen, Hilden, Germany) according to the manufacturer’s instructions.

### 2.2. DNA amplification and sequencing

Partial segments of *nad*5 and *cyt*b genes were amplified to investigate population diversity. In addition to this, β-tubulin 1 (β-*tub*1), β-tubulin 2 (β-*tub*2), and β-tubulin 3 (β-*tub*3) gene isoforms amplification was carried out to determine benzimidazole resistance. The stock solution of the primers was made by adding the mentioned volume of the double deionized water whereas the working solutions of the forward as well as reverse primers were prepared by adding 10 μL of the stock solution to the 90 μL of the ultra-pure water in a 1.5 mL microcentrifuge tube. The forward and reverse sequences of the primers are mentioned in Table 1. The reaction mixture in the PCR tube was comprised of forward and reverse primers (10 pmol), Premix (12.5 μL), sample DNA (0.5 μL), and ultrapure PCR water to make up the final volume of 25 μL. The PCR conditions for amplification of *nad*5, *cyt*b, *tub*1, *tub*2, and *tub*3 genes are given in Table 2.

**Table 1:** The sequence of primers used in this study to amplify full-length *cyt*b, *nad*5, and β-tubulin gene isoforms of *Echinococcus granulosus*.

Primer name	Sequence (5′ - 3′)	Reference
*nad*5 Forward	GTTGTTGAAGTTGATTGTTTTGTTTG	(12)
*nad*5 Reverse	GGAACACCGGACAAACCAAGAA	
*cyt*b Forward	GTCAGATGTCTTATTGGGCTGC	(13)
*cyt*b Reverse	TCTGGGTGACACCCACCTAAATA	
β-*tub*1 Forward	GGATTGCTCTCAGCTTTGAAAACTA	(14)
β-*tub*1 Reverse	CACGGTACTGTTGACTGCCACGACT	
β-*tub*2 Forward	CCTCCAGGGCTTCCAGCTCACCCAC	
β-*tub*2 Reverse	GAAGTGCAGGCGCGGGAATGGAACC	
β-*tub*3 Forward	TTTAGCAGGTGACCAGCCCTTCTAA	
β-*tub*3 Reverse	GAGGTTCGACTGGCGAGGGGCGCAA	

**Table 2:** PCR reaction conditions to amplify partial *cyt*b, *nad*5, β-*tub1*, β-*tub2*, and β-*tub3* genes of *Echinococcus granulosus.*

Steps	Cycles	*cytb*	*Nad5*	*Tub1*	*Tub2*	*Tub3*
Initial denaturation	1	7 min at 95°C				
Denaturation	35	15 s at 95°C	15 s at 95°C	30 s at 95°C	30 s at 95°C	30 s at 95°C
Annealing		40 s at 55°C	30 s at 54°C	30 s at 60°C	30 s at 63°C	30 s at 63°C
Extension step		90 s at 72°C	60 s at 72°C	60 s at 72°C	60 s at 72°C	60 s at 72°C
Final extension	1	7 min at 72°C				

### 2.3. Gel electrophoresis

TAE buffer (1%) was prepared by adding 20 mL of the TAE buffer (50X) to 980 mL of distilled water. Agarose gel (2% w/v) was prepared by adding 1.2 gm of the agarose powder (Agarose, Tsingke Biotechnology Company, Beijing) to the 60 mL of TAE buffer (1%) in a flask. It was allowed to melt in the refrigerator for 2–3 min until it became transparent. Three microliters of the GelRed™ (Biotium, Fremont, United States) were added for the purpose of staining. The gel was poured into the gel casting tray and allowed to solidify. The gel was then placed in the gel electrophoresis apparatus and 5 μL of each amplicon were loaded. A DNA ladder with a length of 2,000 base pairs were run to determine the size of each amplicon.

The gel was run in a gel electrophoresis apparatus (Bio-Red) at 110 V for 20 min. Amplicon (5 μL) in each well of the gel were observed under a UV illuminator. Amplicons were sent to Lanzhou Veterinary Research Institute, China, for sequencing and further analyzes.

### 2.4. Molecular analysis

Sequences were viewed for any misread nucleotide by using MEGA software (v11.0). Corrections in nucleotide sequence and multiple sequence alignment were made through the same software. NCBI-BLAST was used for sample specific sequences to identify them as belonging to a particular species. Sequences were downloaded from the NCBI in FASTA format in order to make the phylogenetic tree. *Taenia hydatigena* was used to make an outgroup. Diversity indices were estimated using DnaSP (v.6) software while phylogenetic analysis was inferred using the Bayesian method using MrBayes (v.1.1) software.

## 3. Results

**Figure 1:**
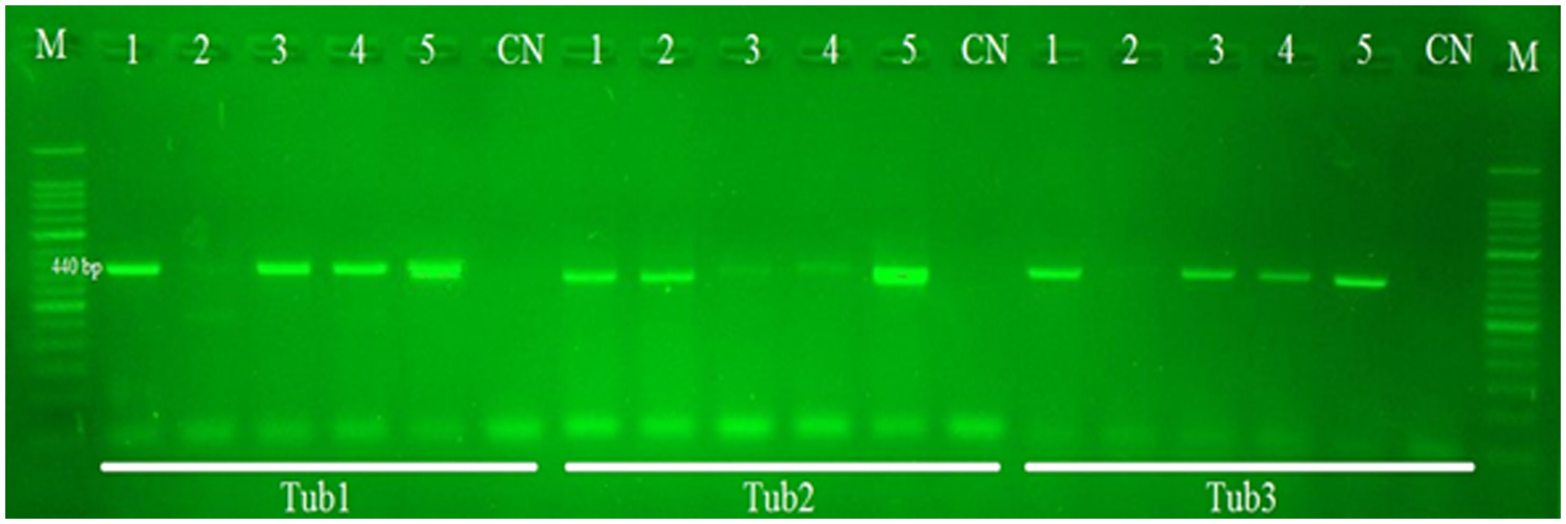
PCR gel results of *Echinococcus tub*1, *tub*2, and *tub*3 partial gene (440 bp) amplification. Lane M contains the DNA marker (50 bp). Lane CN indicates the negative control. Lanes 1–5 indicate positive as well as negative samples from cattle and buffalo.

All 40 samples were found positive for *E. granulosus* when they were subjected to amplification through PCR using *nad*5 and *cyt*b partial genes. The *nad*5 and *cyt*b partial genes generated PCR products of about 680 bp and 580 bp, respectively. β-tubulin gene isoforms (β**-***tub*1, β-*tub*2, and **-***tub*3) produced PCR products of 440 bp as shown in Figure 1.

All PCR products for both of the partial genes were sent for sequencing. After trimming, alignment, and correction of misread nucleotides, the final length of sequences was 625 bp (*nad*5) and 393 bp (*cyt*b) with a total length of 1,018 bp. The aligned and edited sequences created a consensus sequence which was compared to sequences archived in the NCBI database using NCBI BLAST-N.^1^ The sequences were deposited in the GenBank with the accession numbers (ON241337-ON241340) and (ON921008-ON921011) for both *nad5* and *cytb* genes. In this study, only one genotype (G1) of *E. granulosus* was identified.

All the samples were collected from the lungs and livers of cattle and buffaloes; although other organs were also observed for the presence of cysts, samples were only found in those two kinds of organs (lung and liver). It was identified that livers (70%) were more commonly infected compared with lungs (30%). The majority of the samples were infertile. Regarding fertile cysts, 50 % of cysts were of liver origin whereas the remaining fertile samples were collected from the lungs. Comparative data on cyst location and cyst fertility is given in Table 3.

**Table 3:** Data on cyst fertility and organ location in cattle and buffaloes.

Cyst location	Cyst fertility	No. of cyst in cattle	No. of cyst in buffaloes
Liver	Infertile	11	15
Liver	Fertile	1	1
Lung	Infertile	6	4
Lung	Fertile	2	0
Total		20	20

Sequences of nucleotides and their respective encoded amino acids were examined for mutations. A total of seven and six nucleotide mutations were found in *nad*5 and *cyt*b genes, respectively. These resulted in amino acid changes at six and five positions in *nad*5 and *cyt*b genes, respectively.

Nucleotide variation positions of *nad5* and *cytb* genes and their respective amino acids changes are mentioned in Tables 4–7. Positions at which no variation was identified are marked with dot marks whereas a letter indicates a variant at this position. Overall, nucleotide mutations in *nad5* and *cytb* genes were found at seven and six different positions, respectively. Amino acids substitution in *nad5* and *cytb* genes were identified at six and five different sites, respectively.

**Table 4:** *Echinococcus granulosus* mitochondrial *nad*5 gene nucleotide sequence mutation sites.

	2	8	19	83	257	336	572
**Seq1**	C	T	T	A	C	G	T
**Seq2**	.	.	C	.	.	.	.
**Seq3**	T	C	.	.	T	C	C
**Seq4**	.	.	.	G	.	.	.

**Table 5:** *Echinococcus granulosus* mitochondrial *nad*5 gene substitution in amino acids among haplotypes from bovines.

	1	3	7	28	86	191
Seq1	S	V	S	N	P	I
Seq2	.	.	P	.	.	.
Seq3	F	A	.	.	L	T
Seq4	.	.	.	S	.	.

**Table 6:** *Echinococcus granulosus* mitochondrial *cytb* gene nucleotide sequence mutation sites.

	14	19	98	100	107	114
Seq1	G	G	C	A	C	A
Seq2	.	.	.	G	.	.
Seq3	.	.	.	.	.	.
Seq4	A	A	A	.	A	C

**Table 7:** *Echinococcus granulosus* mitochondrial *cytb* gene substitution in amino acids among haplotypes from bovines.

	5	7	33	34	36
Seq1	R	E	A	N	A
Seq2	.	.	.	D	.
Seq3	.	.	.	.	.
Seq4	Q	N	E	.	E

### 3.1. Nucleotide polymorphism and population indices

The diversity of nucleotides and neutrality indices for *E. granulosus* population were calculated on the basis of *nad*5 and *cyt*b partial genes. We observed high haplotype diversity (Hd) and low diversity of nucleotides (π) for *E. granulosus* for both genes in large ruminants (cattle and buffalo). Overall, haplotype diversity (Hd) and nucleotides diversity (π) values were as follow: *nad5* (Hd = 1.00, *π* = 0.00560) and *cytb* = (Hd = 0.833, *π* = 0.00763). The Fu’s Fs values were non-significant (*p* > 0.05) for the *nad*5 gene and *cyt*b genes. The values of Tajima’s D were insignificant and negative for the whole population (Table 8).

**Table 8:** Diversity and neutrality indices for *Echinococcus granulosus* (s.s.) populations from Pakistan based on *nad*5 and *cytb* genes.

Indices	*nad*5 (625 bp)	*cyt*b (393 bp)
No. of isolates	40	40
No. of mutations	7	6
Parsimony informative sites	0	1
No. of haplotypes	4	3
Haplotype diversity (Hd)	1.000	0.833
Nucleotide diversity (π)	0.00560	0.00763
Tajima’s D (*p*-value)	−0.81734	−0.80861
Fu’s Fs (*P*-value)	−1.012	0.731

### 3.2. Phylogenetic analysis

**Figure 2:**
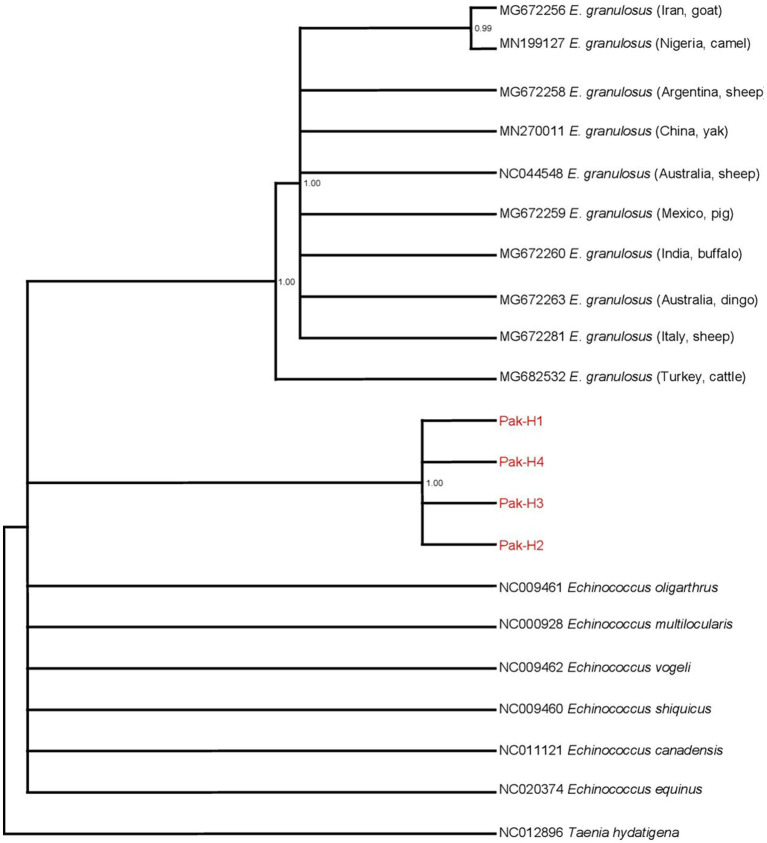
Bayesian phylogeny of Pakistani *E. granulosus* isolates inferred from the *nad*5 and *cytb*. *Taenia hydatigena* was used as an outgroup. Red font indicates *E. granulosus* haplotypes isolated in this study. Posterior probability values are depicted at the nodes.

The *nad*5 main haplotype showed 99% homology with the Turkish isolates from cattle, Italian isolates from sheep, Australian isolates from the dingo, Indian isolates from buffaloes, Mexican isolates from pigs, and Chinese isolates from the yak. Likewise, the *cyt*b main haplotype showed 99% similarity with the Indian isolates from buffaloes, Mexican isolates from pigs, Turkish isolates from sheep, Iranian isolates from cattle, and Argentinian isolates from sheep. Two phylogenic trees were constructed based on *nad*5 and *cyt*b partial genes by combining our submitted sequences with the deposited sequences from other regions in the GenBank. All of the *E. granulosus* (s.s.) isolates from Pakistan were grouped in the same clusters with the relevant reference strain sequences from GenBank, according to a Bayesian phylogeny relying on data of *nad*5–*cyt*b gene sequences. The genotype/species status as distinct from other *Echinococcus* species was validated by Bayesian phylogenetic inference. Pakistani isolates were grouped together and formed a separate cluster while the isolates of other countries formed another cluster (Figure 2). *Taenia hydatigena* (NC012896) was used as an outgroup.

### 3.3. β-tubulin gene isoforms analysis

**Figure 3:**
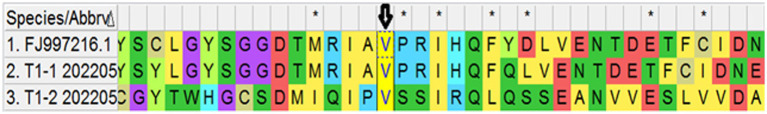
Predicted amino acid sequence of two conformers of Tubulin I. Arrows indicate the valine (V) at position 167.

**Figure 4:**
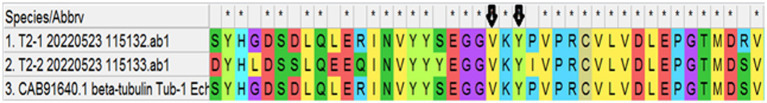
Predicted amino acid sequence of two conformers of Tubulin II. Arrows indicate the valine (V) and tyrosine at position 165 and 167.

**Figure 5:**
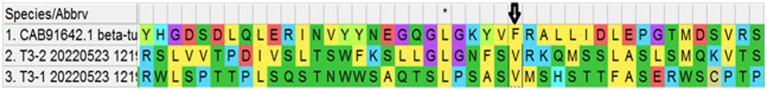
Predicted amino acid sequence of two conformers of Tubulin III. Arrow indicates the valine (V) at position.

After the alignment of the three β-tubulin gene isoforms through MEGA (v. 11.0) software, sequences were compared with the reference sequences from GenBank of *E. multilocularis* in order to identify amino acid substitutions. The accession number of the reference sequences for β-tubulin isoforms I, II, and III were FJ997216, CAB91640, and CAB91642, respectively. The mutation was identified in β-tubulin gene isoform I (position 167), isoform II (position 165 and 167), and isoform III (position 200) as shown in Figures 3–5, respectively.

## 4. Discussion

Infectious diseases including parasitic infestations are important health problems in both animals and humans, which cause economic losses and severe illness (15–20). Parasites are responsible for causing diseases that lead to heavy economical losses in terms of decreased productivity and illness (21–24).

CE is a neglected livestock and public health issue in the world. A high prevalence of this disease can be seen in some areas of China, Central Asia, Peru, and Africa (25). The prevalence is high in those rural/peri-urban areas where aged livestock animals are preferred for slaughtering (26). The reasons behind the presence of disease include lack of awareness and poor hygiene practices (27). However, researchers are trying to change the situation by diagnosing the disease using advanced assays and identifying the extent of drug resistance with the aid of molecular tools.

Since CE is caused by different *Echinococcus* genotypes (G1, G3), therefore, molecular investigations help in better characterization of these genotypes. Identification of the genotype responsible for causing the disease aids in devising preventive and control strategies in a particular geographical area. A limited number of studies on *E. granulosus* have been done in Pakistan up to now which indicates that the disease is underestimated in this region.

Still, government abattoirs continue to play an important role in slaughtering animals in several developing countries including Pakistan, offering economy of scale by putting together livestock owners, processors, and purchasers. Surprisingly, the majority of CE prevalence investigations have been done at urban or peri-urban government abattoirs, implying that such facilities might also exacerbate disease transmission (28). The factors augmenting the disease transmission comprise lack of cooperation by the butchers, poor inspection methods of the veterinary staff, and improper disposal of the infected viscera (29). The current analysis included samples from the main abattoir in Sialkot where people bring cattle and buffalo from different rural, urban, and peri-urban areas with different geographical conditions. This city has a significant population of stray dogs around abattoirs and butcher shops which act as definitive hosts in CE dissemination. Poor hygienic conditions and waste management make the environment favorable for disease transmission to livestock as well as humans. Dogs become infected by eating the viscera (liver and lungs) of diseased animals dumped by the butchers around the slaughterhouse. In this way, the life cycle of the parasite is completed. The same observations were also highlighted previously (30).

Travel to or from prevalent areas and domestic or occupation-based exposure to canids are the risk factors of CE (31, 32). Humans and animals are infection through parasite eggs. The areas around the abattoirs and butcher shops are the main risk factor. The lifestyles of people in rural areas increases the chances of disease transmission where humans and animals live in close vicinity by sharing residence boundaries. Sanitary conditions are very poor not only in rural areas but also in urban areas. All these risk factors contribute to the spread of disease between humans and animals. Therefore, strategies should be devised to control these factors of disease transmission.

Mitochondrial DNA has a significant role in studying intraspecific differences and population genetics due to maternal inheritance, conserved nature, lack of reassortment, high mutation, and evolutionary rate (33–35). Nuclear genes are not preferred due to their different recombination at each generation. In this study, we revealed the population structure of *E. granulosus* (G1/G3) cyst samples collected from cattle and buffalo from the main abattoir in the Sialkot district, Pakistan, based on the partial sequences of two mitochondrial genes, *nad*5 and *cyt*b. The correct identification and characterization of genotypes G1 and G3 is of great epidemiological concern because of the dominance of these two genotypes in animals and humans all over the world.

Although many mitochondrial DNA markers were devised for the molecular identification of *E. granulosus* (36–42), it is controversial to differentiate the genotypes G1 and G3. The *nad5* gene has been considered more reliable in differentiating differences between G1 and G3 genotypes because it possesses six important positions in the comparatively small mitochondrial DNA fragment of 680 bp (12). The *cyt*b gene fragment contains three informative positions. Therefore, we used *nad5* along with the *cyt*b gene in order to explain in a better way the population structure of the *E. granulosus* s.s. complex. Moreover, Kinkar et al. (12) also explained that considering single nucleotide polymorphism in genotype is not sufficient for the discrimination of genotypes G1 and G3 and multiple positions are important to consider. In the previous studies in Pakistan, the population structure of *E. granulosus* in cattle and buffalo was assessed using other genes like *cox*1, *nad*1, 12S *rRNA*, and *rrnL* (ribosomal RNA larger subunit) (4, 43–46), but *nad*5 and *cyt*b genes were not used. In our study, we used short DNA fragments with various informative sites as using larger mitochondrial DNA fragments is not effective or appropriate when the quality of isolated DNA is poor. It is also in accordance with the study by Kinkar et al. (12), who preferred using small mitochondrial DNA fragments with multiple positions. Furthermore, Kinkar et al. (12) suggested that it is better to use the *nad*5 gene along with other markers, which also supports our study since we have used *nad*5 with the *cyt*b gene marker.

Regassa et al. (47) reported that *E. granulosus* mostly infects the liver, lungs, kidneys, heart, and spleen of cattle and buffalo. In this study, all the organs of large ruminants were carefully inspected but only the liver and lungs were found infected with CE which is in accordance with the study conducted by Alvi et al. (48). It was identified that livers (70%) were more commonly infected compared with lungs (30%), which was also verified by Varcasia et al. (49). In contrast to these results, Regassa et al. (47) declared that most samples were of lung origin due to dilatation of lung capillaries in cattle of older age. This might allow the oncosphere to directly pass into the lungs. It could also be carried via thoracic ducts to the lungs and heart and, in this way, the lungs of cattle are more prone to infection compared with the liver.

In this study, it was reported that the lungs and liver have an equal chance of possessing fertile cysts because 50% of cysts were of liver origin whereas the remaining fertile samples were collected from the lungs. In contrast to these results, Regassa et al. (47) reported that the lungs were more commonly identified with fertile cysts compared with the liver.

In this study, genotype G1 was found in all isolates and was confirmed as the most dominant genotype, which is in accordance with the reports from other countries: China (96%), Iran (87%), Brazil (11%), Italy (72%), Turkey, (66%), and Pakistan (44%) (4, 50–54). Moreover, in contrast to current results, genotype G3 was identified as the most prevalent strain as reported by Sharma et al. (55) and Pednekar et al. (56). Thus, it can be concluded that G1 has emerged as the most dominant genotype in East Asia.

In the present study, low nucleotide and high haplotype diversity in the case of both genes revealed the expansion of a small parasite population as previously reported by Sharma et al. (55). A variety of statistical approaches were devised to evaluate the nucleotide neutrality index and population growth (57). The *cyt*b main haplotype showed 99% similarity with the Turkish isolates from sheep, Iranian isolates from cattle, and Argentinian isolates from sheep (12, 58). This finding provides support to the theory of diffusion of CE which postulates that the *E. granulosus* s.s. emerged in the Middle East due to the domestication and farming of sheep and then spread to European, African, American, and Asian countries through the human-related movement of animals (intermediate host) via livestock trading activities (39, 59).

In the current investigation, four samples on the basis of the *nad*5 partial mitochondrial gene yielded four haplotypes, whereas four isolates of the *cyt*b partial gene yielded three haplotypes and were dissimilar to the six haplotypes yielded from 16 isolates collected in a previous study conducted by Alvi et al. (48) and nine haplotypes obtained from 121 isolates as reported by Mehmood et al. (46) using the *nad*5 partial gene. Overall, our findings suggest the occurrence of intraspecies diversity inside *E. granulosus* (s.s.) complex and a growing population in the study region. Variations in haplotype distribution among different populations of *E. granulosus* in Faisalabad and other regions of Pakistan have been reported (48). This might be due to the use of partial sequences (60), investigations on different mitochondrial genes, or the differences in the rate of evolution and mutation of different mitochondrial genes (61). The bovine isolates were identified as *E. granulosus* (G1) by the phylogenetic analysis of the sequenced genes, which grouped closely with the species of *E. granulosus* complex (G1-G10) sequences recovered from the GenBank. All Pakistani isolates formed a separate distinct cluster within the phylogenetic tree, indicating that these haplotypes are different from those of other countries.

In the current study, we used Tajima’s D and Fu’s Fs values to find the trend in population growth. Tajima’s D is a test that compares the overall allelic probability of segregating nucleotides (62). A negative value of the test suggests a tendency toward an excess of uncommon alleles, which is an indication of the recent growing population. The alleles/haplotype distribution is the basis of Fu’s Fs test, and negative results can suggest an overage of alleles, as is being predicted from recent population expansion (63). In the present study, Tajima’s D values were negative and insignificant in the neutrality test based on both genes, showing an increase in low-frequency polymorphism and indicating a significant population expansion. For combined *nad*5 + *cyt*b sequences, Fu’s Fs test values were negative, also indicating a recent population expansion.

The development of resistance in the parasites against different chemical drugs has led to making more efforts to explore alternative or complementary medicines in order to overcome the emerging problem of drug resistance development (64–67). Benzimidazoles are anti-helminth drugs, extensively used all over the world against the helminths of ruminants as well as humans (68). The occurrence of resistance would be assessed phenotypically by considering the poor efficacy of the veterinary medications used to combat parasitic illnesses. This is frequently addressed by increasing the antiparasitic dose rate. Moreover, greater withdrawal times following anthelmintic administration would be required in such instances, but they would not always be maintained, resulting in higher drug residues in human-consumed products which could be a health issue. Anthelmintic resistance allele identification is critical for understanding the mechanisms involved and the epidemiology of anthelmintic resistance. The lack of data on these aspects of disease may put livestock and human populations at high risk and may result in a high burden of disease in the future.

The process of benzimidazole resistance in cattle and buffalo helminths, including *E. granulosus*, has been explored, suggesting that the difference in three different amino acids of the B-tubulin isotype II is involved. Amino acid residue at position 200, 167, or 165 of the gene sequences can result in the development of resistance. Previous studies suggested that phenylalanine (F) amino acid at position 167 and 200 of β-tubulin isoforms indicates benzimidazole sensitivity, whereas the presence of other amino acids such as valine (V) and tyrosine (Y) indicate resistance to benzimidazoles in parasites such as *Haemonchus contortus* and *Teladorsagia circumincta* (69, 70).

A review of the literature suggests that mutations in any one of the three codons (200, 167, and 198) can cause resistance to benzimidazoles, and genotypes analysis of β-tubulin genes of individual helminths revealed that combined genetic changes could not take place in the same allele of β-tubulin isotype-1 protein. This demonstrates that several mutations in the same gene/allele can result in death of the parasite (71, 72). In the current study, benzimidazole resistance was identified in β-tubulin isoforms I at position F165V, isoform II at position F165V and F167Y, and isoform III at position F200V, whereas Pan et al. (10) reported resistance against benzimidazoles in only β-tubulin isoform II at position F165V and F167Y. The results revealed that β-tubulin gene isoforms I, II, and III are responsible for benzimidazole resistance in the selected study area. Efforts should be oriented toward discovering nanoparticle-based antiparasitic drugs as these have shown promising *in-vitro* results (73, 74).

The presence of the zoonotic G1 genotype in cattle and buffalo is of great public health importance, particularly in those areas where the meat of large ruminants is being consumed. Therefore, proper hygienic measures including the disposal of infected viscera should be implemented. This is the first study from Pakistan reporting benzimidazole resistance in *E. granulosus* isolates. The recent findings imply that resistant parasites have been selected during the repeated treatment with benzimidazole and the resistant parasite load will increase if control measures are not optimized. Moreover, investigations should also be carried out in other areas of Pakistan as well to find out the infective parasitic species and status of benzimidazole resistance using the same genes. However, the current study was carried out in one district (Sialkot) and only 40 isolates from cattle and buffaloes were processed for investigations, which are potential limitations of the study.

## 5. Conclusion

The study demonstrates the preponderance of the G1 genotype in bovines slaughtered in the study area. More epidemiological research should be conducted in Pakistan’s various climate zones using additional *Echinococcus* isolates from definitive hosts as well as all intermediate hosts. The molecular analysis of the *nad*5 and *cytb* genes showed a high degree of genetic variation among the Pakistani *E. granulosus* population. These findings on the genetic variation of *E. granulosus* will constitute useful baseline information for future studies on the prevalence and population structure of *E. granulosus* throughout the world based on *nad*5 gene sequences. The study also provided the first molecular evidence of benzimidazole resistance from Pakistan in *E. granulosus* isolates using β-tubulin isoforms that will also help to devise strategies for preventing the spread of benzimidazole resistance globally.

## Data availability statement

The datasets presented in this study can be found in online repositories. The names of the repository/repositories and accession number(s) can be found in the article/Supplementary material.

## Ethics statement

The Ethical approval/consent from the animal owners was not required for the study as no living animal was included in the study and the samples for this study were taken from the condemned carcasses of the slaughtered animals.

## Author contributions

MA, HBY, and MuS conceptualized the study. MA, MS, RA, RB, and MG designed the methodology. MA, RA, LL, YYL, and MSS carried out the validation. MA, RA, and SH wrote the original draft. AA, MI, MUI, HBY, BQF, and WZJ reviewed and edited the manuscript. MA, RA, MuS, and IA revised the manuscript. HBY and WZJ acquired the funding. All authors have read and agreed to the published version of the manuscript.

## Funding

The study received funding from the National Key Research and Development Program (2022YFC2304000), Cultivation of Achievements of State Key Laboratory of Veterinary Etiological Biology (SKLVEB2020CGPY01), Central Public-Interest Scientific Institution Basal Research Fund (Y2022GH13; 1610312020016), and NBCITS (CARS-37).

## Conflict of interest

The authors declare that the research was conducted in the absence of any commercial or financial relationships that could be construed as a potential conflict of interest.

## Publisher’s note

All claims expressed in this article are solely those of the authors and do not necessarily represent those of their affiliated organizations, or those of the publisher, the editors and the reviewers. Any product that may be evaluated in this article, or claim that may be made by its manufacturer, is not guaranteed or endorsed by the publisher.

## Footnotes

^1^ http://blast.ncbi.nlm.nih.gov
