# Supplementary figures and images for: Revealing novel cytb and nad5 genes-based population diversity and benzimidazole resistance in Echinococcus granulosus of bovine origin
Source: Front Vet Sci. 2023 Jun 16;10:1191271. doi: 10.3389/fvets.2023.1191271 (PMC10312306; doi:10.3389/fvets.2023.1191271)

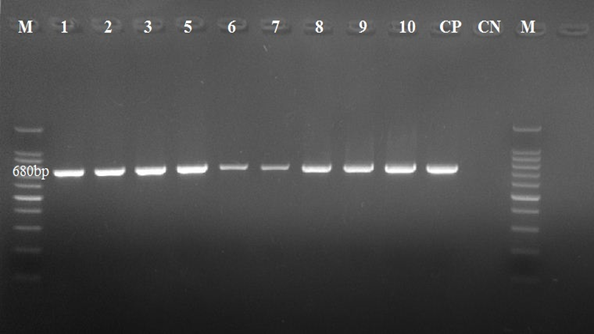

Supplement: Supplementary file 1 [file Image_1.TIF]

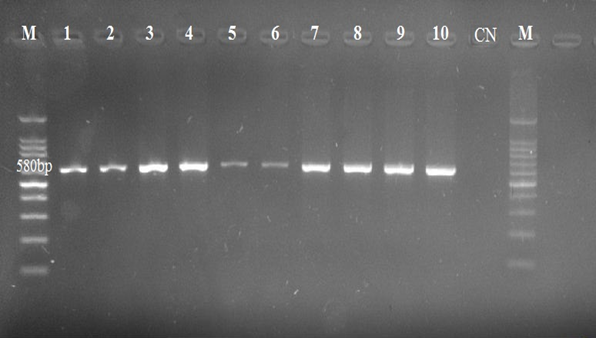

Supplement: Supplementary file 2 [file Image_2.TIF]
